# Modestly Increased Incidence of Irritable Bowel Syndrome in Hidradenitis Suppurativa: A Retrospective Cohort Study Using TriNetX

**DOI:** 10.1111/ijd.70357

**Published:** 2026-02-26

**Authors:** Sahithi Talasila, Areeba Ahmed, Hamza Malick, Umer Nadir, Dario Kivelevitch

**Affiliations:** ^1^ Transitional Year Residency, Orange Park Hospital Florida USA; ^2^ Department of Medicine University of Louisville Louisville Kentucky USA; ^3^ Division of Dermatology Baylor University Medical Center Dallas Texas USA; ^4^ Texas A&M College of Medicine Dallas Texas USA; ^5^ Clarity Dermatology Dallas Texas USA

**Keywords:** epidemiology, gut–skin axis, hidradenitis suppurativa, inflammatory bowel disease, irritable bowel syndrome, retrospective cohort study

Hidradenitis suppurativa (HS) is a systemic disease with a strong association with inflammatory bowel disease (IBD) [1]. However, the relationship between HS and irritable bowel syndrome (IBS), a functional gastrointestinal disorder without underlying structural or inflammatory pathology, remains unclear [2]. One prior study (*n* = 160 patients) found IBS in 67.5% of HS patients and 28.8% of controls, but large‐scale analyses are lacking [2]. Therefore, we designed a retrospective cohort study to evaluate IBS risk in HS patients using a real‐world, multicenter database.

Using the TriNetX Research Network, we conducted a retrospective cohort analysis evaluating adults (≥ 18) with and without HS. We queried codes from the 10th revision of the International Classification of Diseases (ICD‐10) from October 14, 2015 to October 14, 2025 for HS (L73.2). IBS was defined as ≥ 2 ICD‐10‐CM recorded diagnoses for K58, a definition which has been previously validated [3]. Patients with IBD, celiac disease, microscopic colitis, colorectal cancer, cystic fibrosis, short bowel syndrome, small intestinal bacterial overgrowth, chronic pancreatitis, chemotherapy, or a history of major bowel surgery, at any point in their medical record, were excluded. Additionally, patients with IBS prior to their documented HS diagnosis were excluded. Lastly, patients with infectious gastroenteritis within 30 days prior to the study were excluded. Propensity score matching (1:1) was performed on age, demographics, and relevant comorbidities, including obesity, diabetes mellitus, tobacco use, gastroesophageal reflux disease, functional dyspepsia, anxiety, depression, systemic antibiotic use, proton pump inhibitor use, and chronic opioid use (Table 1). We assessed risk ratios (RRs) and absolute risk differences (ARDs) for IBS at 1, 5, and 10 years after HS diagnosis. Statistical significance was defined as a *p*‐value < 0.05.

**TABLE 1:** Baseline demographic and clinical characteristics of patients with hidradenitis suppurativa (HS) and patients without HS after propensity score matching (PSM).

	Baseline characteristics before PSM			Baseline characteristics after PSM		
	HS cohort (*n* = 205,340)	No HS cohort (*n* = 666,754)	*p*‐value	HS cohort (*n* = 129,031)	No HS cohort (*n* = 129,031)	*p*‐value
Age at index, Mean ± Standard Deviation	37.7 ± 13.8	56.7 ± 16.7	< 0.0001	41 ± 14.3	41 ± 15.4	0.52
Male	56,302 (27.4%)	276,998 (41.6%)	< 0.0001	46,274 (35.9%)	46,180 (35.8%)	0.93
Female	146,982 (71.6%)	382,245 (57.3%)	< 0.0001	80,692 (62.5%)	80,823 (62.6%)	0.91
White	110,684 (53.9%)	478,237 (71.7%)	< 0.0001	80,512 (62.4%)	80,431 (62.3%)	0.92
Black or African American	63,655 (31.0%)	93,346 (14.0%)	< 0.0001	9077 (21.4%)	9123 (21.6%)	0.77
Asian	5416 (2.6%)	22,336 (3.4%)	0.002	3924 (3.0%)	3887 (3.0%)	0.88
Hispanic or Latino	20,623 (10.0%)	59,829 (9.0%)	0.01	12,333 (9.6%)	12,284 (9.5%)	0.93
Not Hispanic or Latino	183,709 (89.5%)	604,416 (90.6%)	0.01	116,031 (89.9%)	116,086 (90.0%)	0.93
Overweight and obesity	69,771 (34.0%)	147,082 (22.1%)	< 0.0001	37,247 (28.9%)	37,316 (28.9%)	0.96
Diabetes mellitus	36,954 (18.0%)	86,732 (13.0%)	< 0.0001	18,694 (14.5%)	18,598 (14.4%)	0.91
Smoking status	51,335 (25.0%)	120,016 (18.0%)	< 0.0001	9499 (22.4%)	9457 (22.3%)	0.94
Gastroesophageal reflux disease	34,908 (17.0%)	80,010 (12.0%)	< 0.0001	18,059 (14.0%)	17,991 (13.9%)	0.95
Anxiety	51,074 (24.9%)	107,214 (16.1%)	< 0.0001	24,923 (19.3%)	24,815 (19.2%)	0.85
Depression	57,433 (27.9%)	106,687 (16.0%)	< 0.0001	28,053 (21.7%)	28,135 (21.8%)	0.88
Systemic antibiotics	33,121 (16.1%)	65,674 (9.8%)	< 0.0001	15,669 (12.1%)	15,728 (12.2%)	0.9
Proton pump inhibitors	26,418 (12.9%)	63,950 (9.6%)	< 0.0001	12,776 (9.9%)	12,733 (9.9%)	0.93
Chronic opioid use	22,716 (11.1%)	44,249 (6.6%)	< 0.0001	9278 (7.2%)	9211 (7.1%)	0.87

We identified 129,031 HS patients and 129,031 matched controls. At 1 year follow‐up, the RR of IBS in HS patients was 1.13, with an ARD of 0.078% (*p* = 0.0134). By 5 years, RR was.

1.16, with ARD 0.23% (*p* < 0.0001) (Table 2). Lastly, at 10 years RR 1.17, with ARD 0.29% (*p* < 0.0001). Despite increased relative risk, ARDs translate to ~1, 2, or 3 excess cases per 1000 HS patients at 1, 5, and 10 years. These findings highlight the importance of interpreting relative risk estimates within the context of absolute risk and that statistical significance does not necessarily equate to meaningful clinical impact.

**TABLE 2:** Risk of irritable bowel syndrome (IBS) development in patients with hidradenitis suppurativa (HS) at 1, 5, and 10 years.

Outcome	Risk ratio (95% CI^a^), 1 year	Absolute risk difference (95% CI), 1 year	*p*‐value	Risk ratio (95% CI), 5 years	Absolute risk difference (95% CI), 5 years	*p*‐value	Risk ratio (95% CI), 10 years	Absolute risk difference (95% CI), 10 years	*p*‐value
IBS	1.13 (1.03–1.25)	0.078% (0.016–0.14)	0.013	1.16 (1.07–1.23)	0.23% (0.13–0.33)	< 0.0001	1.17 (1.11–1.24)	0.29% (0.19–0.40)	< 0.0001

^a^ CI: Confidence Interval.

Therefore, clinicians should be aware of this potential risk but interpret it cautiously, considering both the modest ARDs observed and the broader context of the patient's clinical presentation and comorbidities. This distinction between statistical and clinical significance is crucial to avoid overstating the relevance of relative risk alone. Nevertheless, the biological plausibility of this relationship is supported by evidence that HS and IBS may share common gut–skin axis mechanisms. These include intestinal dysbiosis, immune dysregulation with elevated proinflammatory cytokines, and overlapping metabolic and signaling pathways influenced by diet [4, 5].

Limitations of our study include its retrospective design and reliance on ICD‐10 coding to identify IBS, a functional diagnosis that introduces the potential for diagnostic misclassification. Additional misclassification may have occurred due to symptomatic overlap between IBD and IBS, including early or atypical IBD presentations not yet formally diagnosed despite lifetime exclusion criteria, as well as the stronger established association of HS with IBD and the higher prevalence of IBS in the general population [2]. Residual confounding is also possible, as psychosocial stressors, socioeconomic status, dietary factors, and duration of medication exposures could not be directly measured or controlled within the TriNetX database. Further, patients with HS may have increased healthcare utilization and greater exposure to medications such as antibiotics, proton pump inhibitors, and opioids, potentially introducing surveillance or detection bias that increases the likelihood of IBS diagnosis despite matching for these variables.

## Funding

The authors have nothing to report.

## Disclosure

No artificial intelligence (AI) or large language models were used in the writing, data analysis, figure/table creation, or editing of this manuscript. All text and analyses were conducted by the authors.

## Ethics Statement

Data accessible via TriNetX are presented in aggregate form and only contain anonymized data as per the deidentification standard defined by the US Health Insurance Portability and Accountability Act (HIPAA) in Section 164,514(a). Given this study used only de‐identified data and did not involve individually identifiable patient data, this study was exempt from Institutional Review Board Approval.

## Conflicts of Interest

The authors declare no conflicts of interest.

## Data Availability

The data that support the findings of this study are available from TriNetX. Restrictions apply to the availability of these data, which were used under license for this study. Data are available from the author(s) with the permission of TriNetX.
